# On the entry of an emerging arbovirus into host cells: Mayaro virus takes the highway to the cytoplasm through fusion with early endosomes and caveolae-derived vesicles

**DOI:** 10.7717/peerj.3245

**Published:** 2017-04-27

**Authors:** Carlos A.M. Carvalho, Jerson L. Silva, Andréa C. Oliveira, Andre M.O. Gomes

**Affiliations:** 1 Programa de Biologia Estrutural, Instituto de Bioquímica Médica Leopoldo de Meis, Universidade Federal do Rio de Janeiro, Rio de Janeiro, Brazil; 2 Instituto Nacional de Ciência e Tecnologia de Biologia Estrutural e Bioimagem, Universidade Federal do Rio de Janeiro, Rio de Janeiro, Brazil; 3 Current address: Seção de Arbovirologia e Febres Hemorrágicas, Instituto Evandro Chagas, Ministério da Saúde, Ananindeua, Pará, Brazil

**Keywords:** Mayaro virus, Arboviruses, Virus entry, Endocytosis, Clathrin-coated vesicles, Caveolae-derived vesicles

## Abstract

Mayaro virus (MAYV) is an emergent sylvatic alphavirus in South America, related to sporadic outbreaks of a chikungunya-like human febrile illness accompanied by severe arthralgia. Despite its high potential for urban emergence, MAYV is still an obscure virus with scarce information about its infection cycle, including the corresponding early events. Even for prototypical alphaviruses, the cell entry mechanism still has some rough edges to trim: although clathrin-mediated endocytosis is quoted as the putative route, alternative paths as distinct as direct virus genome injection through the cell plasma membrane seems to be possible. Our aim was to clarify crucial details on the entry route exploited by MAYV to gain access into the host cell. Tracking the virus since its first contact with the surface of Vero cells by fluorescence microscopy, we show that its entry occurs by a fast endocytic process and relies on fusion with acidic endosomal compartments. Moreover, blocking clathrin-mediated endocytosis or depleting cholesterol from the cell membrane leads to a strong inhibition of viral infection, as assessed by plaque assays. Following this clue, we found that early endosomes and caveolae-derived vesicles are both implicated as target membranes for MAYV fusion. Our findings unravel the very first events that culminate in a productive infection by MAYV and shed light on potential targets for a rational antiviral therapy, besides providing a better comprehension of the entry routes exploited by alphaviruses to get into the cell.

## Introduction

Virus entry into the host cell is a crucial step of the viral infection cycle and represents the first hijacking of some constitutive cellular function by the parasite. For those viruses that have a surrounding lipid bilayer—the so-called enveloped viruses, the entry process usually involves fusion of the viral envelope with either the plasma membrane or the endosomal membrane of the host cell (Más & Melero, 2013).

Among the enveloped viruses, the mechanism adopted by the members of the genus *Alphavirus* (family *Togaviridae*) during cell entry is well studied, however there are still gaps to be filled. This virus genus includes arboviruses related to either encephalitogenic or arthritogenic diseases in humans and is represented by the prototypes Sindbis (SINV) and Semliki Forest (SFV) viruses (Gould et al., 2010). While SFV entry classically occurs by receptor-mediated endocytosis followed by fusion with the endosomal membrane (Helenius et al., 1980; Marsh, Bolzau & Helenius, 1983), more recent works suggest that SINV delivers its genome in the host cell through a pore-like structure formed at the plasma membrane level without the occurrence of membrane fusion—which is unusual for enveloped viruses (Paredes et al., 2004; Wang et al., 2007).

Despite intense studies on these prototypical alphaviruses, knowledge on several members of the genus is still obscure, even though these viruses represent important public health concern (LaBeaud, 2008). One such example is Mayaro virus (MAYV), an alphavirus endemic in the Amazon region and closely related to Chikungunya virus (CHIKV), which has recently spread in an epidemic way over many areas of the world. Just as with CHIKV, MAYV infection in humans leads to a febrile illness followed by a highly debilitating arthralgia (De Figueiredo & Figueiredo, 2014).

MAYV is transmitted by the same vector which transmits the sylvatic Yellow Fever virus, namely *Haemagogus janthinomys* (Pinheiro & LeDuc, 1998), and it was already shown that, at least in laboratory, MAYV can replicate in and be transmitted by *Aedes aegypti*, an anthropophilic mosquito highly adapted to urban areas (Long et al., 2011). Due to partial overlap of symptoms, Mayaro fever may be misdiagnosed as Dengue fever and other exanthematous febrile diseases, thus underestimating the actual number of human cases of MAYV infection (Zuchi et al., 2014). The increasing number of outbreaks of Mayaro fever in South America (Lwande et al., 2015) coupled with its constant import to non-endemic areas as far as Europe (Receveur et al., 2010; Hassing et al., 2010; Neumayr et al., 2012; Theilacker et al., 2013; Slegers et al., 2014), reinforce the imminent threat of MAYV urban emergence. Despite the relevance of MAYV as a human pathogen and the potential for its emergence in urban areas, Mayaro fever is yet a greatly neglected disease (Forshey et al., 2010).

In this work, we investigated the early events of MAYV infection by tracking the virus entry into the host cell and the interactions of virus particles with different cell compartments. Our results provide new insights on the mechanism used by alphaviruses to invade the target cells.

## Materials & Methods

### Cell culture

MAYV (VR-1277, genotype D) was obtained from the American Type Culture Collection (Manassas, VA, USA). Baby hamster kidney (BHK-21) and African green monkey kidney (Vero) cells were cultured as monolayers in 25-cm^2^ flasks (TPP, Trasadingen, Switzerland) at 37 °C in a humidified atmosphere with 5% CO_2_ in DMEM (Sigma-Aldrich, St. Louis, MO, USA) supplemented with 10% fetal bovine serum (Cultilab, Campinas, SP, Brazil) and 5 µg/mL gentamicin sulfate (Invitrogen, Carlsbad, CA, USA).

### Virus propagation and purification

MAYV was propagated and purified as previously described (Mezencio & Rebello, 1993) with several modifications. BHK-21 cells were grown to quasi-confluence in 300-cm^2^ flasks (TPP, Trasadingen, Switzerland) and then infected with MAYV under a multiplicity of infection (MOI) of 0.1 plaque-forming unit/cell (PFU/cell) for 48 h at 37 °C. After virus propagation, the culture supernatant was collected and cleared of cell debris by centrifuging at 8,000 rpm for 20 min at 4 °C in an RPR 12-2 rotor (Hitachi, Tokyo, Japan). The supernatant was applied to a 30% sucrose cushion and centrifuged in a Type 45 Ti rotor (Beckman Coulter, Brea, CA, USA) at 32,000 rpm for 1 h 40 min at 4 °C. The pellet was suspended in PBS, layered onto a discontinuous 5–50% sucrose density gradient and centrifuged at 30,000 rpm for 1 h 30 min at 4 °C in an SW 40 Ti rotor (Beckman Coulter). Fractions were collected, and the fraction containing the virus was identified by reading the optical density at 260 and 280 nm. Purified virions were aliquoted and stored at −80 °C until further use.

### Fluorescent labeling of virus particles

Approximately 10^10^ MAYV particles—based on the protein content of the virus preparation, as determined by the Lowry method (Lowry et al., 1951)—were incubated with 2 nmol of DiD (Molecular Probes, Eugene, OR, USA) in PBS for 10 min at room temperature. The unincorporated dye was removed by centrifuging through an Amicon Ultra filter unit with a 100-kDa molecular weight cut-off (Millipore, Billerica, MA, USA). Labeled virus particles were suspended in PBS, passed through a syringe-driven filter unit with 0.22-µm pore size to remove virus aggregates and immediately used for experiments. DiD labeling was confirmed by scanning the light absorption spectrum of the virus sample. For double-labeling, virus sample was incubated with 50 µM SYTO 82 (Molecular Probes) for 1 h at room temperature before adding DiD.

### Assays for virus infection efficiency

Vero cell monolayers in 12-well plates (TPP, Trasadingen, Switzerland) were pre-treated with 500 µL of dansylcadaverine, chlorpromazine, ammonium chloride, chloroquine or methyl-β-cyclodextrin at the indicated concentrations in DMEM for 1 h at 37 °C. After this pre-treatment step, the medium containing the compounds was removed and cells were incubated with 100 µL of MAYV at 100 PFU/well for 1 h at 37 °C while maintaining the respective concentrations of the above chemicals (except for methyl-β-cyclodextrin, which was incubated with the cells only prior to virus addition). Subsequently, the medium containing non-adsorbed virus particles was replaced by a semisolid medium (1.6% carboxymethylcellulose in DMEM) supplemented with 2% fetal bovine serum and 5 µg/mL gentamicin sulfate. After incubation for 48 h at 37 °C, cells were simultaneously fixed and stained with a solution composed of 1% crystal violet, 30% ethanol, and 7.4% formaldehyde in PBS to allow for plaque counting. The efficiency of virus infection under different treatment conditions was determined by comparing the number of plaques formed in treated cells with that formed in untreated cells, the latter being regarded as 100% of infection.

For the analysis of fluorophore incorporation effect on virus infectivity, MAYV was first labeled with DiD, serially diluted and then subjected to plaque assay in Vero cells. After incubation for 48 h at 37 °C, cells were fixed and stained as above. Virus titer was determined by plaque counting and expressed as a ratio to the volume.

### Cell transfection with plasmid vectors

Transfection of Vero cells with the vectors pDsRed-Rab5WT, pGFP-Rab7WT or pGFP-Cav1 (Addgene, Cambridge, MA, USA) was performed using the FuGENE HD Transfection Reagent (Promega, Madison, WI, USA) according to the manufacturer’s instructions. Approximately 5 × 10^4^ cells at subconfluence were incubated with 0.2 µg of the plasmid of interest in Opti-MEM I (Invitrogen) under a transfection reagent:DNA ratio of 3:1 (in the case of co-transfection with two vectors, the transfection mixture contained 0.1 µg of each plasmid) for 1 h at 37 °C. After this time, the medium was replaced by DMEM supplemented with 2% fetal bovine serum and 5 µg/mL gentamicin sulfate and the cells were then incubated for 18 h at 37 °C to allow for vector expression.

### Imaging of virus entry and fusion with endocytic vesicles

Subconfluent Vero cells seeded in 35-mm glass-bottom dishes (MatTek, Ashland, MA, USA) were used for all imaging procedures. To image virus entry, cells were incubated with DiD-labeled MAYV under the indicated MOI for 15 min at 4 °C. After this virus binding synchronization step, unbound virus particles were washed away with PBS, and cells were incubated in DMEM supplemented with 2% fetal bovine serum and 5 µg/mL gentamicin sulfate at 37 °C to allow for virus entry. At the indicated times after temperature raising, cells were washed again with PBS and fixed with 3.7% formaldehyde for 15 min. When so stated, cells were previously transfected with the vectors pDsRed-Rab5WT, pGFP-Rab7WT or pGFP-Cav1. Samples were visualized on an LSM 510 META laser-scanning confocal fluorescence microscope (Carl Zeiss, Oberkochen, Germany) with excitation at 633 nm and emission collected from 650 to 710 nm for imaging DiD-labeled virus. When infected with double-labeled MAYV, cells were also excited at 561 nm and emission was collected from 575 to 615 nm for imaging SYTO 82-labeled virus. For imaging DsRed- or GFP-labeled proteins, laser excitation was performed at 561 or 488 nm and emission was collected from 575 to 615 nm or from 500 to 530 nm, respectively. For the time-lapse experiment, cells were kept alive in DMEM without phenol red supplemented as above and the frames were acquired at a rate of 1 fps with the temperatures of both sample stage and objective lens regulated by a TempControl 37-2 Digital heating unit (PeCon, Erbach, Germany) to maintain the culture medium at 37 °C. Images were processed using ImageJ 1.48 software (National Institutes of Health, Bethesda, MD, USA).

### Statistical analyses

Statistical analyses were performed using an unpaired *t*-test with a two-tailed *P*-value and a one-way ANOVA with the Dunnett’s post-test on Prism 6 software (GraphPad Software, San Diego, CA, USA). Data were expressed as the mean ± range or standard deviation (SD), and *P*-values less than 0.05 were considered statistically significant.

## Results

### MAYV enters the host cells through the endocytic pathway

**Figure 1:**
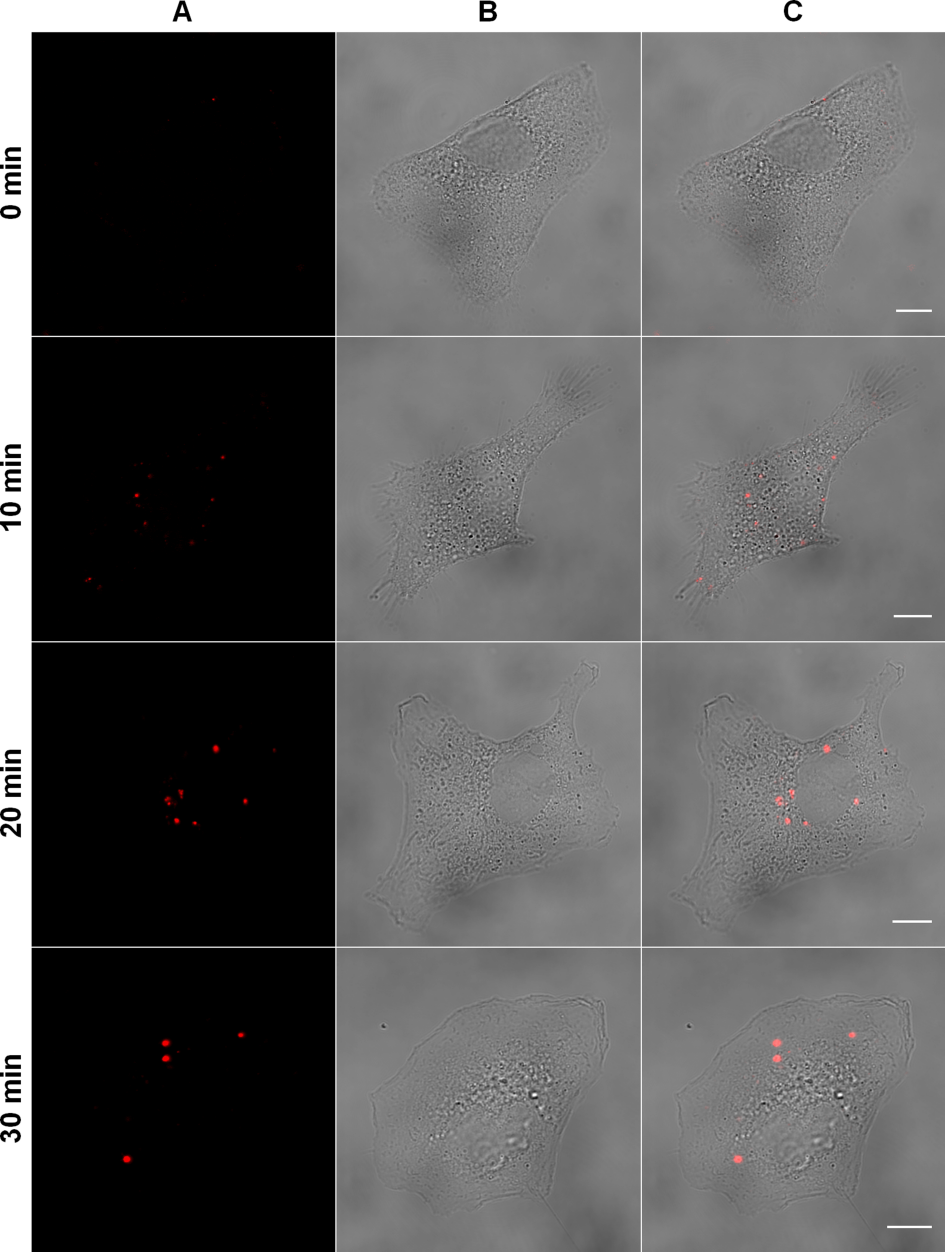
Tracking of MAYV entry into the host cell. Subconfluent Vero cells were infected with DiD-labeled MAYV under an MOI of 100 PFU/cell. Infection was synchronized by first allowing virus binding to cell surface for 15 min at 4 °C (time zero), and then temperature was raised to 37 °C to allow for infection progress. At the indicated times after temperature raising, cells were fixed and visualized by confocal fluorescence microscopy, using excitation at 633 nm and emission from 650 to 710 nm. The selected individual cells are representative of the respective open visual field (*n* = 3). (A) DiD fluorescence channel. (B) brightfield channel. (C) overlapping of fluorescence (DiD) and brightfield channels. Scale bars: 10 μm.

In order to determine the nature of the route taken by MAYV to enter the cell, the envelopes of virus particles were labeled with the lipophilic fluorescent dye DiD. We did not observe any significant difference on infectivity between labeled and unlabeled virus (Fig. S1). The labeling was performed under a high density of DiD molecules per virion so that virus particles were initially almost non-fluorescent due to the characteristic self-quenching of this fluorophore. Upon virus fusion with a cellular target membrane, one could expect the fluorescence intensity to increase specifically at the site of the event as DiD molecules from the donor membrane diluted into the acceptor membrane—thus relieving the fluorescence quenching. On the other hand, no increase in fluorescence intensity should be expected if a non-fusogenic route was the rule. Based on this reasoning, Vero cells were synchronously infected with DiD-labeled MAYV under a high MOI and the fluorescent signals were imaged at different times by fluorescence microscopy after virus binding (Fig. 1).

As expected, low-fluorescent virus particles were observed bound to the cellular plasma membrane by the end of the infection synchronization step at 4 °C. However, after raising the temperature to 37 °C several fluorescent particles moved into the cytoplasm and an increase in fluorescence intensity at 10 min was observed, indicating the occurrence of viral fusion with endosomal membranes. At 20 and 30 min, we observed that post-fusion fluorescent signals moved towards the perinuclear region of the cell and were larger and less numerous.

### Endocytosed MAYV particles are positive for viral RNA

Although endocytosed MAYV particles were shown to trigger a fusion reaction with their uptake vesicles, one could still argue they might be merely empty virus particles that were nonspecifically endocytosed after having delivered their genomes through the plasma membrane.

**Figure 2:**
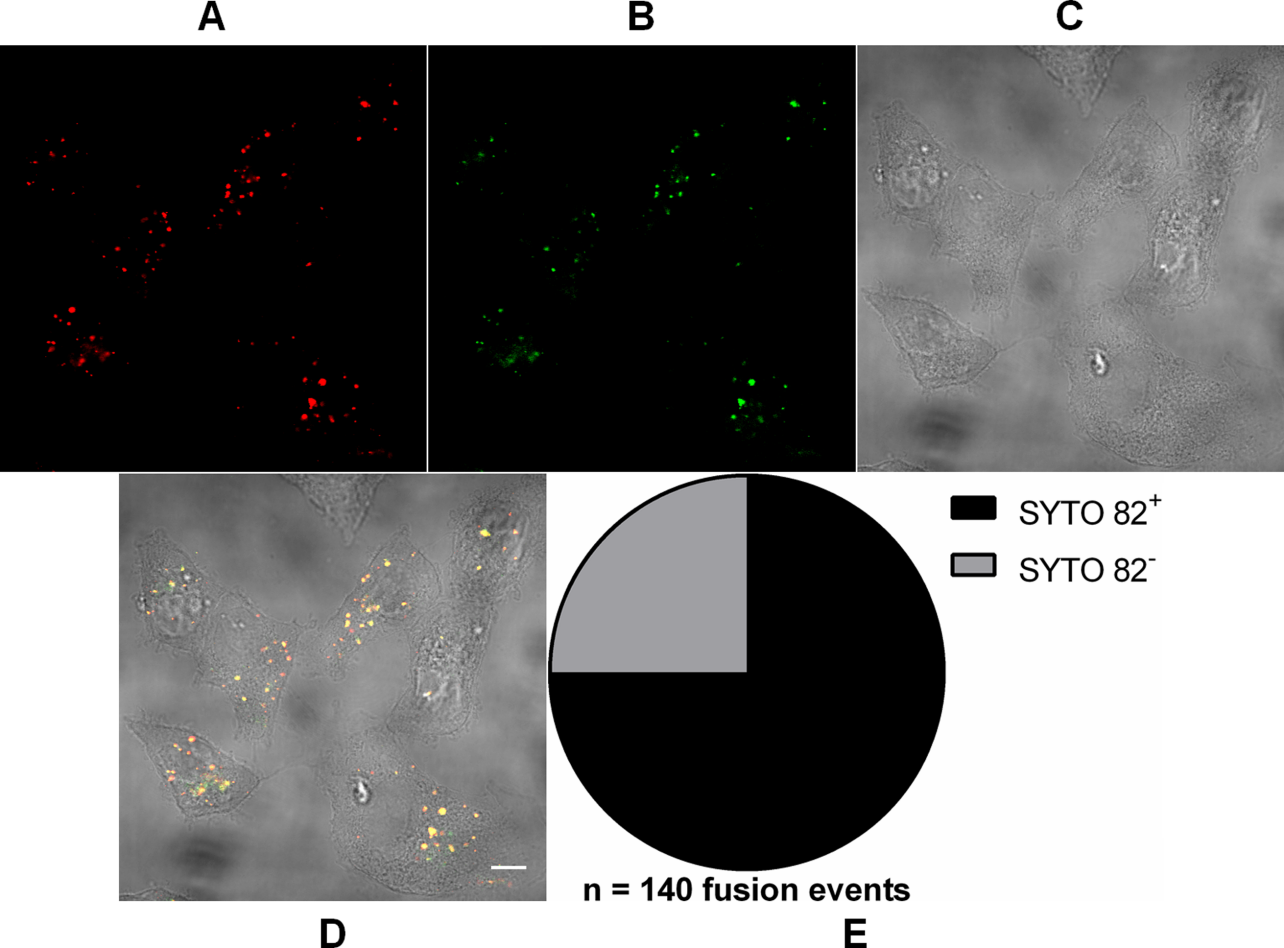
Analysis of RNA presence in endocytosed MAYV particles. Subconfluent Vero cells were infected with MAYV double-labeled with DiD (red) and SYTO 82 (green) under an MOI of 100 PFU/cell. Infection was synchronized by first allowing virus binding to cell surface for 15 min at 4 °C (time zero), and then temperature was raised to 37 °C to allow for infection progress. At 15 min after temperature raising, cells were fixed and visualized by confocal fluorescence microscopy. DiD fluorescence was imaged as described in Fig. 1; SYTO 82 was excited at 561 nm and emission was collected from 575 to 615 nm. Co-localization events of DiD and SYTO 82 signals are shown in yellow. The selected cells are representative of the respective open visual field (*n* = 2). (A) DiD fluorescence channel. (B) SYTO 82 fluorescence channel. (C) brightfield channel. (D) overlapping of fluorescence (DiD and SYTO 82) and brightfield channels. Scale bar: 10 μm. (E) Proportion of fused virus particles which were SYTO 82^+^ or SYTO 82^−^ (*n* = 140 fusion events).

In order to show that the incoming particles were not empty, we previously double-labeled virus particles with DiD and SYTO 82—a non-virucidal RNA-binding fluorescent dye capable of permeating the viral envelope and reaching the viral genome through capsid fenestrations (Brandenburg et al., 2007)—and then imaged the cells at 15 min after viral binding to Vero cell surface (Fig. 2). 75 ± 7.4% (mean ± SD, *n* = 140 fusion events) of DiD fluorescence signals co-localized with those of SYTO 82, indicating that the virus particles contained RNA and that the vast majority of such nucleic acid remains associated with the endocytic compartments after fusion.

### Fusion between MAYV and endosomes occurs soon after virus internalization

**Figure 3:**
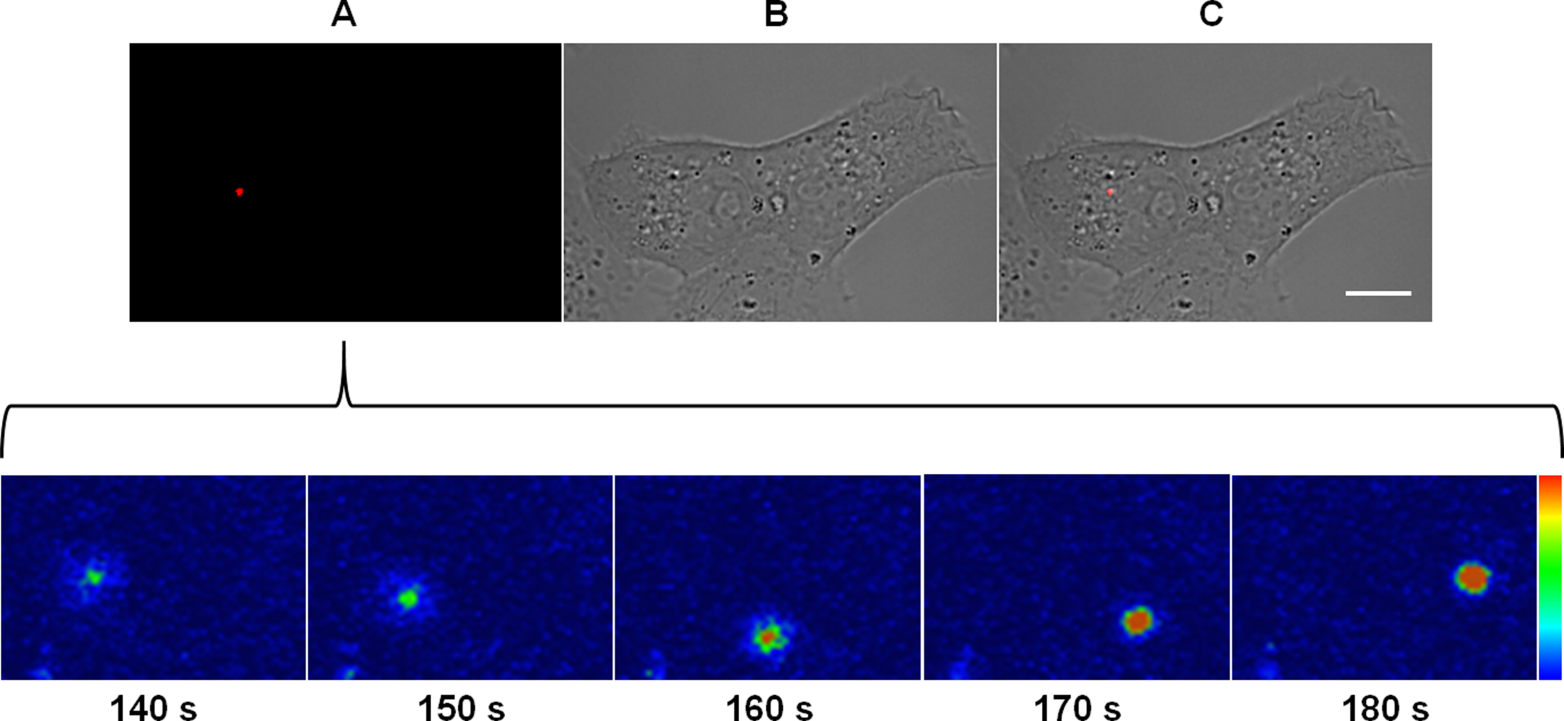
Kinetics of the membrane fusion reaction driven by MAYV. Subconfluent Vero cells were infected with DiD-labeled MAYV under an MOI of 10 PFU/cell. Infection was synchronized by first allowing virus binding to cell surface for 15 min at 4 °C (time zero), and then temperature was raised to 37 °C on the microscope stage to allow for infection progress. Fluorescent signals in individual cells were tracked by time-lapse fluorescence microscopy. Frames show the endocytic traffic behavior of a single virus particle until fusion of its envelope with the endosomal membrane at the selected times after temperature raising. Colored bar indicates the variation of DiD fluorescence intensity from low (blue) to high (orange). The selected individual virus particle is representative of the median time to maximum DiD fluorescence dequenching (*n* = 5). (A) DiD fluorescence channel. (B) brightfield channel. (C) overlapping of fluorescence (DiD) and brightfield channels. Scale bar: 10 μm.

Next, we determined the kinetics of the viral fusion with endosome membrane. To this end, Vero cells were synchronously infected with DiD-labeled MAYV and DiD fluorescence dequenching from single incoming virus particles was then followed by time-lapse fluorescence microscopy (Fig. 3).

As revealed by DiD fluorescence dequenching of a virus particle which represented the median time to reach the maximum fluorescence intensity, the beginning of the fusion between the virus envelope and the endosomal membrane was detected at as early as 2.5 min after the virus had bound to the cellular plasma membrane and took around 30 s to reach the peak of dequenching. Although some virus particles were observed to fuse within 1 or 2 min post-binding, most of the virus fusion events concentrated near 3 min post-binding, varying from 45 to 200 s (Video S1).

### MAYV entry involves clathrin, low pH, and cholesterol

**Figure 4:**
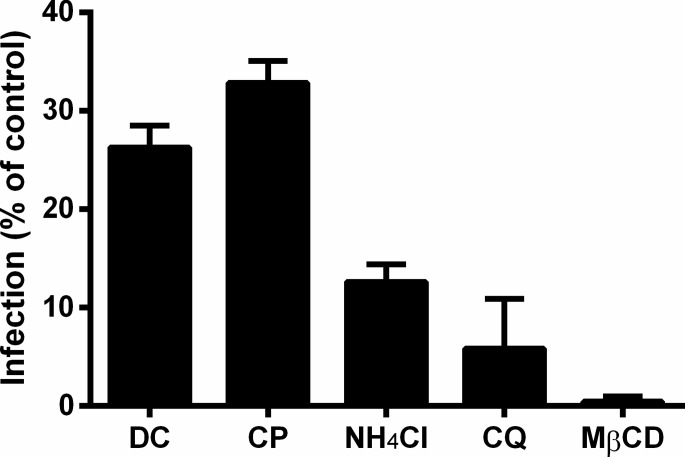
Role of clathrin, low pH, and cholesterol in the initial steps of MAYV infection. Vero cell monolayers were pre-treated with 200 μM dansylcadaverine (DC), 50 μM chlorpromazine (CP), 20 mM ammonium chloride (NH_4_Cl), 20 μM chloroquine (CQ) or 10 mM methyl-β-cyclodextrin (MβCD) for 1 h and then infected with MAYV under the same MOI. The first four chemicals were maintained during the viral adsorption step, while the last one was removed to avoid depletion of viral envelope cholesterol. At 48 h post-infection, cells were fixed and stained with crystal violet to determine the efficiency of infection by comparing plaque counts. All differences compared to the respective controls (infected cells pre-treated with DMEM alone) were significant (*P* < 0.001). Bars: mean ± SD (*n* = 3).

We next investigated the role of clathrin-mediated endocytosis, endosomal low pH and membrane cholesterol in MAYV infection. To this end, Vero cells were previously treated with dansylcadaverine or chlorpromazine (two pharmacological inhibitors of clathrin-mediated endocytosis), ammonium chloride or chloroquine (two weak bases capable of raising the pH of acidic compartments) and methyl-β-cyclodextrin (a cyclic glucose oligomer known to deplete cholesterol from biological membranes), and then infected with MAYV under the same MOI to assess virus plaque formation in each of these conditions (Fig. 4).

While inhibition of clathrin-mediated endocytosis led to an average reduction of 70% (74% by dansylcadaverine and 67% by chlorpromazine) in the viral infection efficiency, raising the pH of acidic compartments and depleting the cholesterol from cellular membranes had effects even more pronounced, that caused average reduction of 91% (87% by ammonium chloride and 94% by chloroquine) and 99% in the viral infection efficiency, respectively.

### Early but not late endosomes are implicated in MAYV fusion

**Figure 5:**
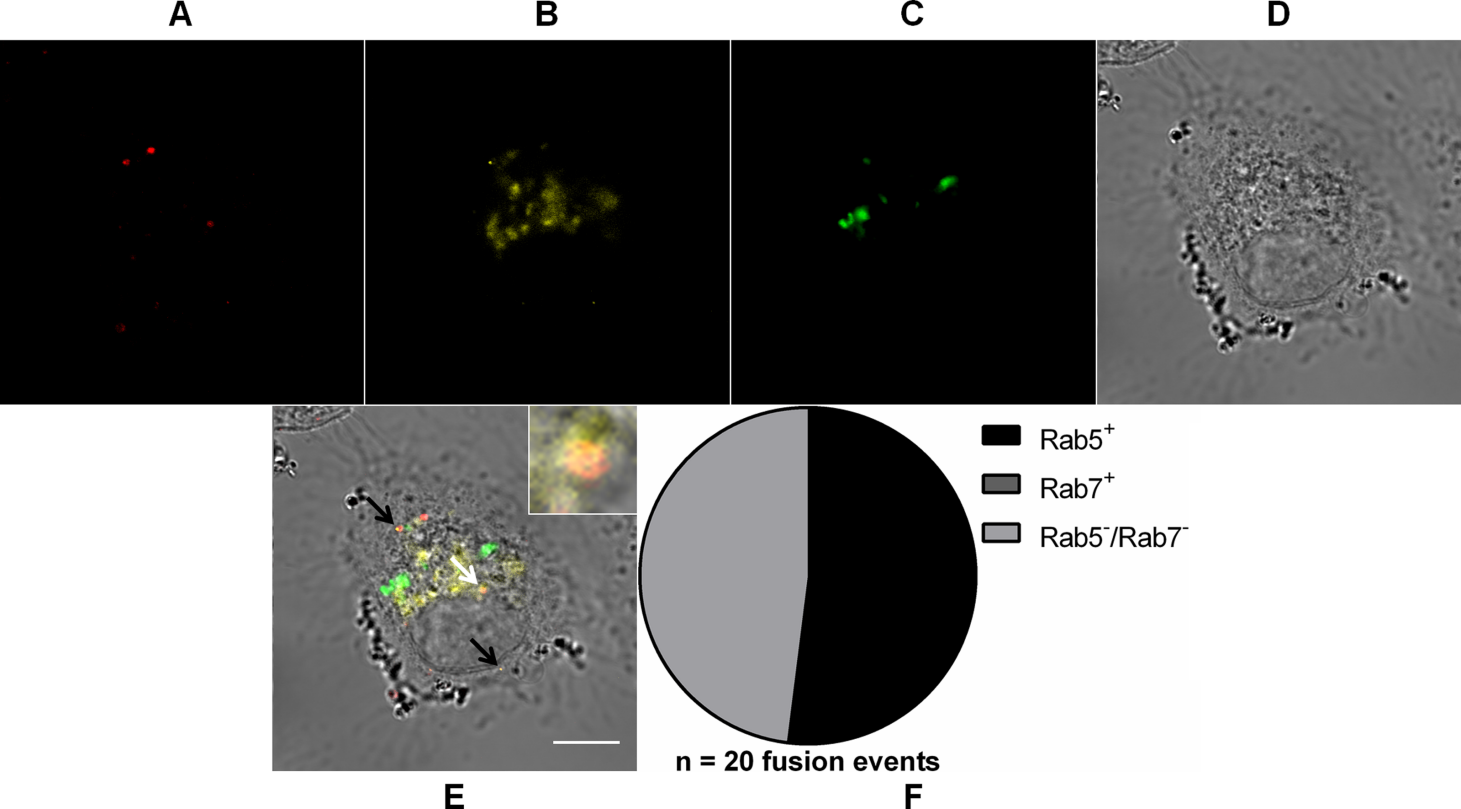
Role of early and late endosomes in MAYV fusion. Subconfluent Vero cells were simultaneously transfected with vectors pDsRed-Rab5WT (yellow) and pGFP-Rab7WT (green) and 18 h later infected with DiD-labeled MAYV (red) under an MOI of 10 PFU/cell. Infection was synchronized by first allowing virus binding to cell surface for 15 min at 4 °C (time zero), and then temperature was raised to 37 °C to allow for infection progress. At 15 min after temperature raising, cells were fixed and visualized by confocal fluorescence microscopy. Arrows point to co-localization events of DiD and DsRed fluorescent signals (orange). The selected individual cell is representative of the population of cells that were both transfected and infected (*n* = 2). (A) DiD fluorescence channel. (B) DsRed fluorescence channel. (C) GFP fluorescence channel. (D) brightfield channel. (E) overlapping of fluorescence (DiD, DsRed, and GFP) and brightfield channels. Inset: magnification of the co-localization event pointed by the white arrow. Scale bar: 10 μm. (F) Distribution of fused virus particles between Rab5^+^, Rab7^+^, and Rab5^−^/Rab7^−^ vesicles (*n* = 20 fusion events).

We used the same strategy as before to track DiD-labeled MAYV particles during cell entry and to identify the endocytic compartment to which MAYV fuses its envelope we used the expression of fluorescent versions of Rab guanosine triphosphatases (GTPases), a class of proteins that regulate transportation, sorting and maintenance of endosomal vesicles along the endocytic pathway and are often partitioned in a specific way in different endosome classes. As the clathrin-mediated endocytosis pathway often delivers the endocytosed cargo to early and then late endosomes, DiD-labeled MAYV was then tracked by confocal fluorescence microscopy in Vero cells transiently expressing both Rab5 (resident in early endosomes) fused to DsRed and Rab7 (resident in late endosomes) fused to GFP (Fig. 5).

We observed that 52 ± 10.4% (mean ± range, *n* = 20 fusion events) of the virus fusion events co-localized with Rab5^+^ vesicles, but not even a single virus fusion event co-localized with Rab7^+^ vesicles—in other words, no co-localization with either early or late endosomes was detected for the remaining of such events.

### MAYV fuses alternatively with caveolae-derived vesicles

**Figure 6:**
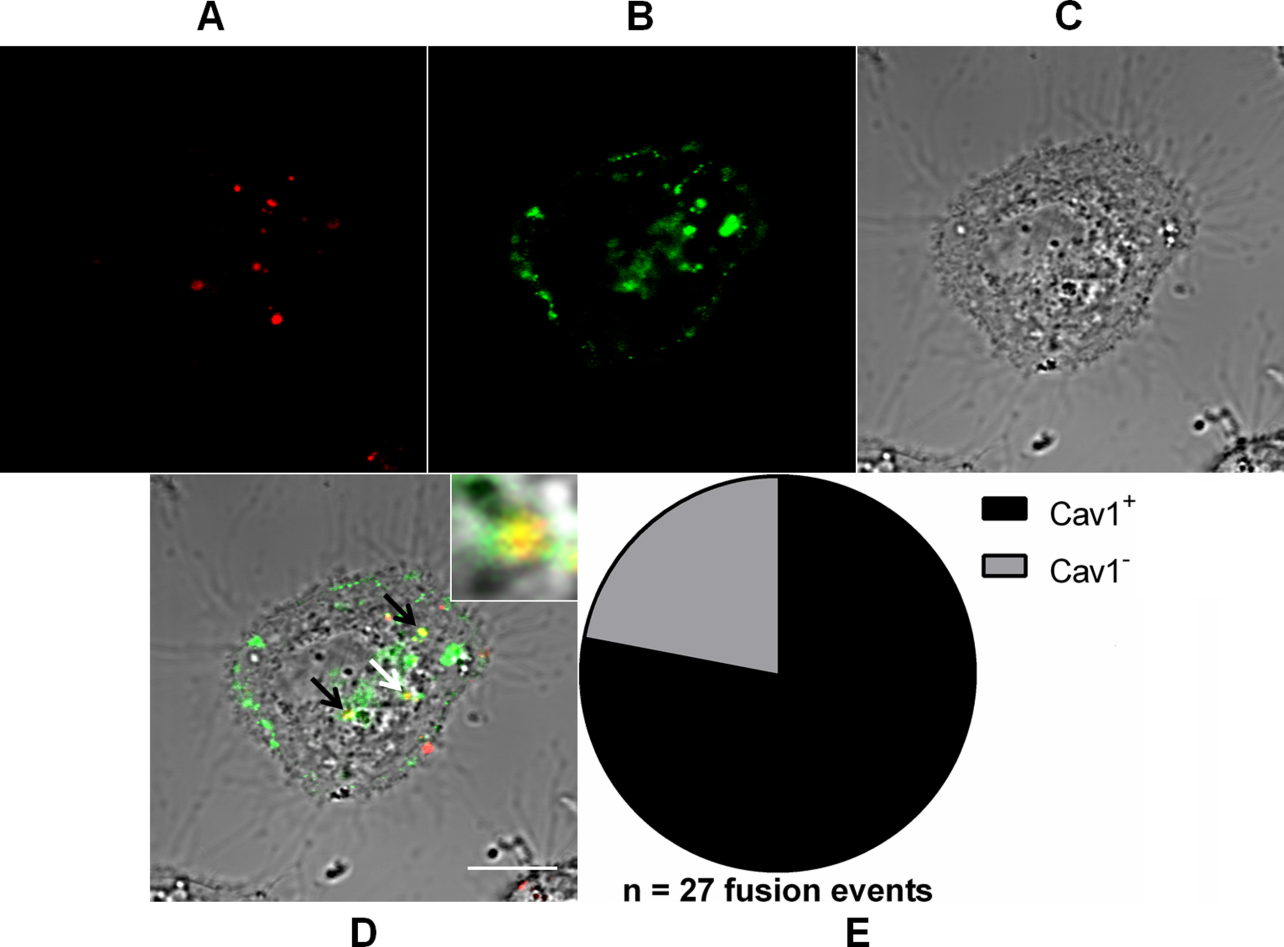
Role of caveolae-derived vesicles in MAYV fusion. Subconfluent Vero cells were transfected with vector pGFP-Cav1 (green) and 18 h later infected with DiD-labeled MAYV (red) under an MOI of 10 PFU/cell. Infection was synchronized by first allowing virus binding to cell surface for 15 min at 4 °C (time zero), and then temperature was raised to 37 °C to allow for infection progress. At 15 min after temperature raising, cells were fixed and visualized by confocal fluorescence microscopy. Arrows point to co-localization events of DiD and GFP fluorescent signals (yellow). The selected individual cell is representative of the population of cells that were both transfected and infected (*n* = 2). (A) DiD fluorescence channel. (B) GFP fluorescence channel. (C) brightfield channel. (D) overlapping of fluorescence (DiD and GFP) and brightfield channels. Inset: magnification of the co-localization event pointed by the white arrow. Scale bar: 10 μm. (E) Distribution of fused virus particles between Cav1^+^ and Cav1^−^ vesicles (*n* = 27 fusion events).

Given the strong dependence on cholesterol for virus entry, we investigated whether the lack of co-localization of some virus-fusion competent compartments with early or late endosome markers could be due to an alternative route for MAYV internalization that involves cholesterol-enriched membrane microdomains, such as caveolae. Similar as before, Vero cells expressing GFP-tagged caveolin-1 (Cav1), which is the main protein structural component of caveolae, were infected with DiD-labeled MAYV, and the fluorescent signals were then tracked by confocal fluorescence microscopy (Fig. 6).

Notably, 78 ± 0.8% (mean ± range, *n* = 27 fusion events) of the virus fusion events co-localized with Cav1^+^ vesicles, suggesting the existence of an important alternative route for MAYV entry into the cell.

### MAYV fusion-competent compartments may intersect in the endocytic pathway

**Figure 7:**
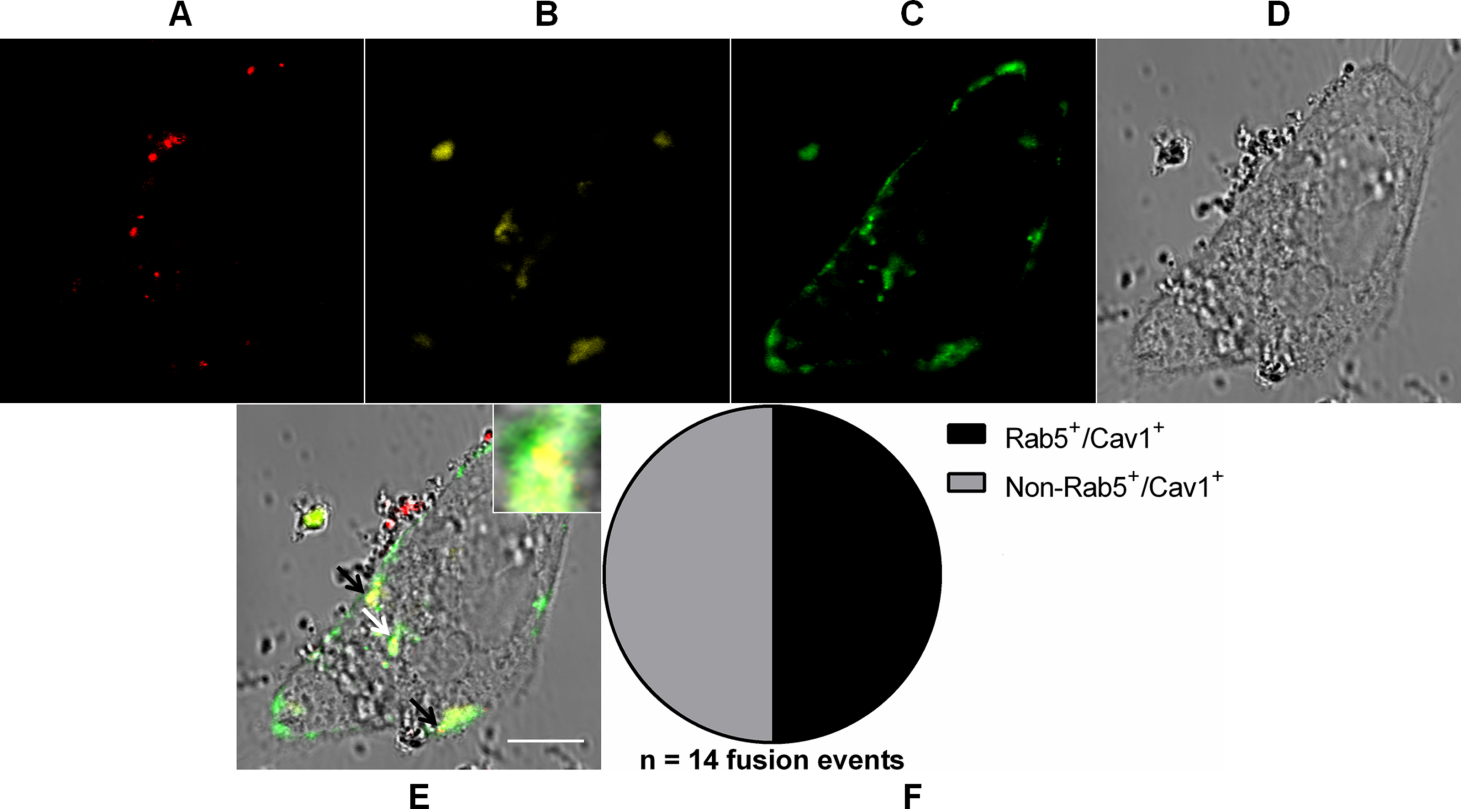
Coalescence of MAYV fusion-competent compartments. Subconfluent Vero cells were simultaneously transfected with vectors pDsRed-Rab5WT (yellow) and pGFP-Cav1 (green) and 18 h later infected with DiD-labeled MAYV (red) under an MOI of 10 PFU/cell. Infection was synchronized by first allowing virus binding to cell surface for 15 min at 4 °C (time zero), and then temperature was raised to 37 °C to allow for infection progress. At 15 min after temperature raising, cells were fixed and visualized by confocal fluorescence microscopy. Arrows point to co-localization events of DiD, DsRed, and GFP fluorescent signals (orange). The selected individual cell is representative of the population of cells that were both transfected and infected (*n* = 2). (A) DiD fluorescence channel. (B) DsRed fluorescence channel. (C) GFP fluorescence channel. (D) brightfield channel. (E) overlapping of fluorescence (DiD, DsRed, and GFP) and brightfield channels. Inset: magnification of the co-localization event pointed by the white arrow. Scale bar: 10 μm. (F) Distribution of fused virus particles between Rab5^+^/Cav1^+^ and non-Rab5^+^/Cav1^+^ vesicles (*n* = 14 fusion events).

Since MAYV was shown to fuse with early endosomes and caveolae-derived vesicles, the distribution of incoming virus particles between these two compartments was analyzed by tracking DiD-labeled MAYV in Vero cells transiently expressing both DsRed-tagged Rab5 and GFP-tagged Cav1 by confocal fluorescence microscopy (Fig. 7).

As expected for the non-complementary relative distribution of virus fusion events between early endosomes and caveolae-derived vesicles assessed before, rather than distinctly distributed between these two endocytic compartments, half the number (*n* = 14 fusion events) of MAYV particles were found fused with vesicles that were both Rab5^+^ and Cav1^+^.

## Discussion

In this work, we show that MAYV entry into Vero cells occurs via the endocytic pathway, which agrees with the classical studies done on the entry of the prototypical alphavirus SFV (Helenius et al., 1980; Marsh, Bolzau & Helenius, 1983). Tracking the fluorescence dequenching of DiD-labeled virions at the single particle level, we observed that MAYV fuses with the endosomal membrane in approximately 3 min after binding to the cell surface. From binding to fusion, this virus takes approximately 3 min to complete this process, while other species such as Influenza virus and Dengue virus (DENV), take approximately 10 min to carry out the same tasks (Rust et al., 2004; Van der Schaar et al., 2007). Our data reveal that MAYV fusogenic reaction occurs within early compartments in the endocytic pathway, which may explain the shorter time taken by MAYV to reach the cytosol when compared to virus species known to fuse with late endosomes (Yoshimura et al., 1982; Van der Schaar et al., 2008). Such fast endosomal escape may represent a selected advantage by avoiding virus particle exposure to highly-active acid hydrolases and allowing an early onset of viral genome replication.

Many single-virus tracking studies have employed lipophilic fluorescent probes to label enveloped viruses in order to analyze the role of membrane fusion during their entry into target cells (Brandenburg & Zhuang, 2007). The choice of fluorophores to be used in such analyses is often driven by their efficiency to be incorporated in the viral envelope without compromising the virus particle infectivity. However, it is important to highlight that fluorophore-labeled viruses are usually inactivated upon exposure to microscope excitation light (Wainwright, 2004). The fact that DiD-labeled viruses do not suffer photoinactivation explains our choice for DiD rather than other lipophilic fluorophores—e.g., octadecyl rhodamine B (R18)—to label MAYV envelope (Thongthai & Weninger, 2009). Given this fact and the characteristic low particle-to-PFU ratio of alphavirus preparations (Hernandez, Brown & Paredes, 2014), our virus tracking setup increases the chances of tracking a virion capable of generating a productive infection. Besides, we observed a strong positive correlation between efficiently-fused and genome-containing particles, which reveals that the internalized viruses are not empty particles that were nonspecifically endocytosed after having delivered their genomes through the plasma membrane, further supporting the infectious potential of our virus preparation.

Interestingly, our particle tracking experiments revealed a decrease in the number of fluorescent spots concomitantly to an increase in their size as a function of the time elapsed after virus internalization, suggesting that the vesicles exploited by MAYV for cell entry converge to a common structure located close to the perinuclear region. Such structure may be the previously described type I cytopathic vacuoles (CPVs-I), on which surface viral genome replication occurs (Froshauer, Kartenbeck & Helenius, 1988; Kujala et al., 2001).

Our analysis of specific requirements during virus entry points clathrin-mediated endocytosis as an important pathway for MAYV entry into the cell. Considering that clathrin-coated vesicles are directed to early endosomes, whose contents are acidified by ATP-powered proton pumps (Goldstein et al., 1985), the findings that MAYV entry is inhibited by acidotropic weak bases and occurs through virus fusion with Rab5^+^ vesicles further support a role for such pathway in virus entry. Regarding the mobilization of early endosomes, the mechanism of MAYV entry is similar to that described for the alphaviruses SFV and CHIKV. Nevertheless, while SFV is internalized in the target cell via clathrin-mediated endocytosis, CHIKV entry is mediated by a process that does not depend on this protein (Vonderheit & Helenius, 2005; Bernard et al., 2010). Our finding showing that only a fraction of the incoming MAYV particles fuses with Rab5^+^ vesicles is in accordance with a previous study on SINV entry into BHK-21cells, which showed that about 40% of the incoming virus particles did not co-localize with Rab5^+^ vesicles (Gu, Yang & Liu, 2011).

**Figure 8:**
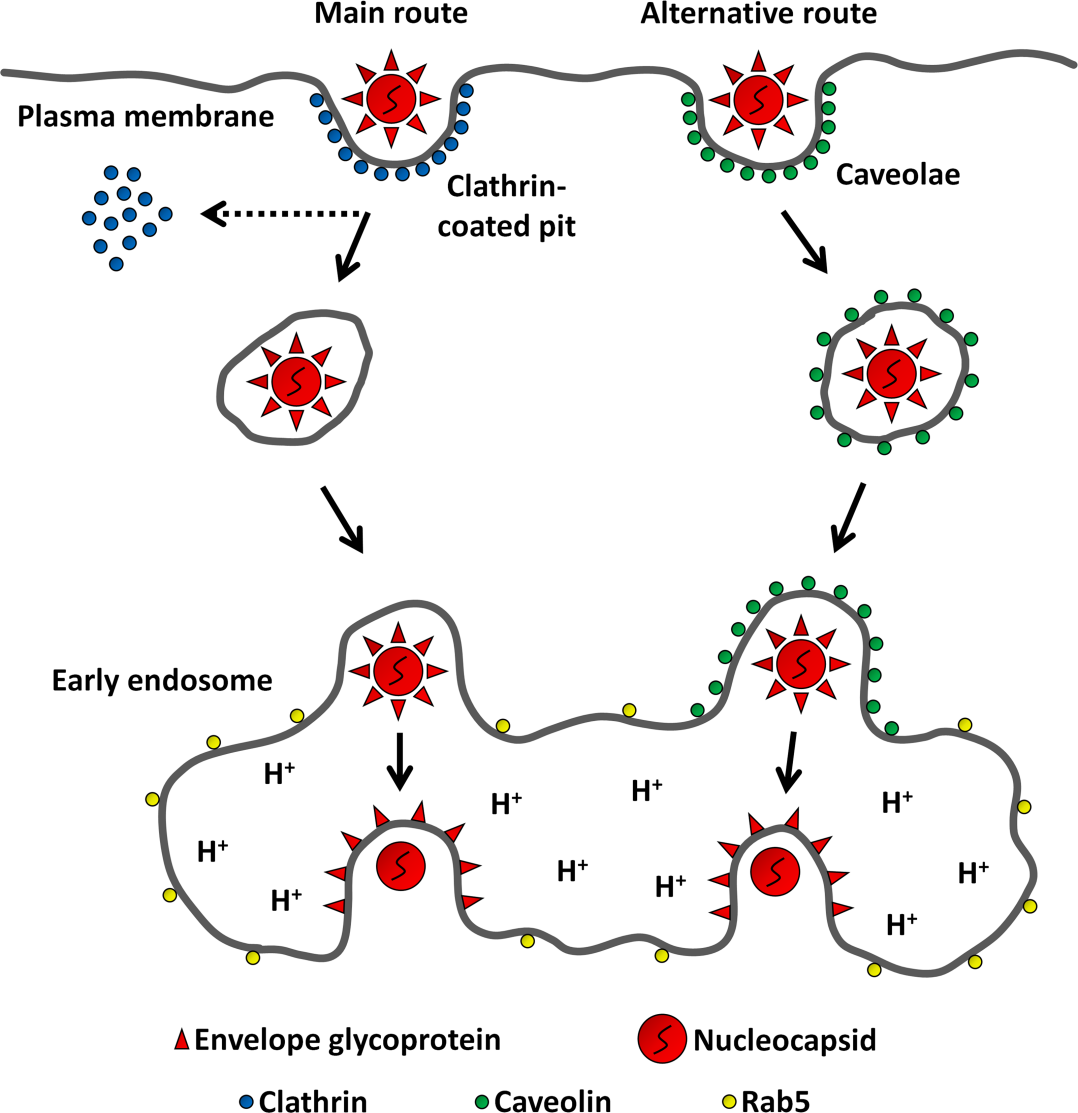
Model for MAYV entry into the host cell. After binding to receptors on the cell surface, MAYV particles take endocytic routes to reach the cytosol. While most virions are internalized via clathrin-coated pits, a minor fraction uses caveolae as portals of entry. Regardless of the internalization pathway, endocytosed viruses are delivered into early endosomes where the membrane fusion reaction occurs triggered by low pH.

Moreover, we show that MAYV alternatively uses cholesterol-enriched caveolae-derived vesicles to get into the cytoplasm, which helps to explain its critical dependence on cholesterol for infection—as a matter of fact, a specific interaction of SINV and SFV glycoproteins with target-membrane cholesterol has been observed before (Kielian, Chanel-Vos & Liao, 2010). It should be noted that MAYV seems to mobilize cholesterol-enriched domains also to exit the host cell, since its envelope bears high-cholesterol content (Sousa Jr et al., 2011). Taken together, these observations suggest that such membrane microdomains are widely exploited during the infection cycle of MAYV. Interestingly, some Cav1^+^ vesicles to which MAYV particles fused were shown to co-localize with Rab5^+^ vesicles, suggesting that virions taking such alternative route are subsequently delivered into early endosomes. Indeed, cargo uptake via caveolae-derived vesicles does not exclude a later association with early endosomes (Pelkmans et al., 2004). Given the strong MAYV dependence on low pH for its entry, this passage through an early endosome may be a critical step for an efficient virus infection (Fig. 8). It is worth highlighting that the caveolae-derived vesicles tracked in our experiment do not represent the formerly proposed “caveosomes”, large neutral pH-endocytic compartments enriched in caveolin whose existence was questioned half a decade ago (Parton & Howes, 2010).

Despite the negative effect of cholesterol depletion by methyl-β-cyclodextrin on caveolae structure, it is worth noting that actin cytoskeleton arrangement is also affected by treatment with the drug (Ao et al., 2016). Such nonspecific effect could compromise membrane trafficking events from cell periphery to the cell interior, including clathrin-mediated endocytosis, thus further contributing to the remarkable deleterious effect of methyl-β-cyclodextrin treatment on MAYV infection and confirming the strong dependence on endocytosis for its internalization. Moreover, although our results clearly support MAYV entry through the endocytic route, exploring different pathways, we cannot rule out the hypothesis that the unusual mechanism of entry through the plasma membrane without the occurrence of membrane fusion, as previously suggested for SINV (Paredes et al., 2004; Wang et al., 2007), may be also used by MAYV. However, given that disturbances of the endocytic pathway strongly inhibit virus infection, we conclude that any non-endocytic route would be of minor significance for MAYV infection.

It is noteworthy that the urbanization of Mayaro fever from the Amazon region is imminent and that the virus has a real potential to adapt to new vectors (Long et al., 2011) and spread to non-endemic areas (Receveur et al., 2010; Hassing et al., 2010; Neumayr et al., 2012; Theilacker et al., 2013; Slegers et al., 2014). If it manages to do so, MAYV may become the next public health burden in the tropical region, following the paths of other arboviruses such as DENV and, more recently, CHIKV and Zika virus (Musso, Cao-Lormeau & Gubler, 2015). Besides providing a better understanding on the endocytic model of alphavirus entry, our findings shed light on potential targets for a future rational therapy against MAYV.

##  Supplemental Information

10.7717/peerj.3245/supp-1 Figure S1 Effect of DiD labeling on MAYV infectivity Purified MAYV particles were incubated with DiD in PBS (+DiD) or with PBS alone (−DiD) for 10 min at room temperature and analyzed for infectivity by plaque assay in Vero cells after removal of the unincorporated dye. After 48 h of infection, cells were stained, and the virus plaques were counted to determine the virus titer. Difference between +DiD and −DiD conditions was not significant (*P* = 0.2626). Bars: mean ±range (*n* = 2).

10.7717/peerj.3245/supp-2 Video S1 Trafficking of incoming MAYV particles until fusion Subconfluent Vero cells were infected with DiD-labeled MAYV under an MOI of 10 PFU/cell. Infection was synchronized by first allowing virus binding to cell surface for 15 min at 4 °C (time zero), and then temperature was raised to 37 °C on the microscope stage to allow for infection progress. Fluorescent signals in host cells were tracked by time-lapse fluorescence microscopy. The video is representative of the endocytic traffic behavior of single virus particles up to 200 s after temperature raising (*n* = 5). Colors indicate the variation of DiD fluorescence intensity from low (red) to high (green) due to fusion of the viral envelope with the endosomal membrane. Scale bar: 10 μm.

10.7717/peerj.3245/supp-3 Supplemental Information 3 Raw data for Fig. 1

10.7717/peerj.3245/supp-4 Supplemental Information 4 Raw data for Fig. 1B

10.7717/peerj.3245/supp-5 Supplemental Information 5 Raw data for Fig. 1C

10.7717/peerj.3245/supp-6 Supplemental Information 6 Raw data for Fig. 1D

10.7717/peerj.3245/supp-7 Supplemental Information 7 Raw data for Fig. 2

10.7717/peerj.3245/supp-8 Supplemental Information 8 Raw data for Fig. 4

10.7717/peerj.3245/supp-9 Supplemental Information 9 Raw data for Fig. 5

10.7717/peerj.3245/supp-10 Supplemental Information 10 Raw data for Fig. 6

10.7717/peerj.3245/supp-11 Supplemental Information 11 Raw data for Fig. 7

10.7717/peerj.3245/supp-12 Supplemental Information 12 Raw data for Fig. S1

## Additional Information and Declarations

### Data Availability

The following information was supplied regarding data availability: The raw data has been supplied as Supplementary Files. Gomes, Andre (2017): Raw data Mayaro virus entry into host cells. Figshare. https://doi.org/10.6084/m9.figshare.4679533.v1.
