# Supplementary figures and images for: On the entry of an emerging arbovirus into host cells: Mayaro virus takes the highway to the cytoplasm through fusion with early endosomes and caveolae-derived vesicles
Source: PeerJ. 2017 Apr 27;5:e3245. doi: 10.7717/peerj.3245 (PMC5410162; doi:10.7717/peerj.3245)

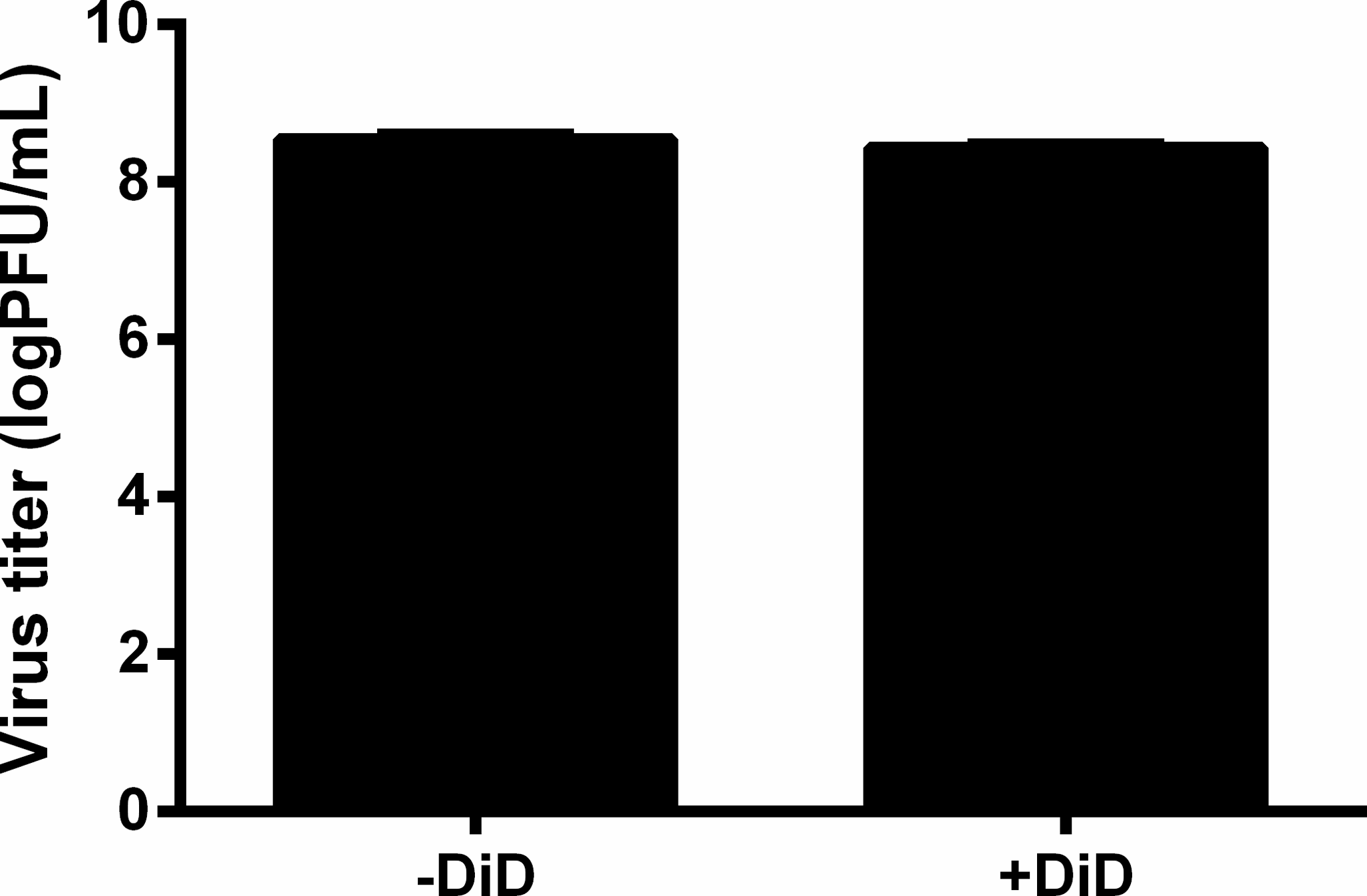

Supplement: Figure S1 — Purified MAYV particles were incubated with DiD in PBS (+DiD) or with PBS alone (−DiD) for 10 min at room temperature and analyzed for infectivity by plaque assay in Vero cells after removal of the unincorporated dye. After 48 h of infection, cells were stained, and the virus plaques were counted to determine the virus titer. Difference between +DiD and −DiD conditions was not significant (P = 0.2626). Bars: mean ±range (n = 2). [file peerj-05-3245-s001.png]
